# RNA Sequencing Keloid Transcriptome Associates Keloids With Th2, Th1, Th17/Th22, and JAK3-Skewing

**DOI:** 10.3389/fimmu.2020.597741

**Published:** 2020-11-23

**Authors:** Jianni Wu, Ester Del Duca, Michael Espino, Alyssa Gontzes, Inna Cueto, Ning Zhang, Yeriel D. Estrada, Ana B. Pavel, James G. Krueger, Emma Guttman-Yassky

**Affiliations:** ^1^ Laboratory of Inflammatory Skin Diseases, Department of Dermatology, Icahn School of Medicine at Mount Sinai, New York, NY, United States; ^2^ College of Medicine, State University of New York Downstate Medical Center, Brooklyn, NY, United States; ^3^ Department of Dermatology, University of Rome Tor Vergata, Rome, Italy; ^4^ Laboratory for Investigative Dermatology, The Rockefeller University, New York, NY, United States; ^5^ Department of Biomedical Engineering, University of Mississippi, Oxford, MS, United States

**Keywords:** RNA-seq, keloids, immune, Th2, JAK3, inflammation

## Abstract

Keloids are disfiguring, fibroproliferative growths and their pathogenesis remains unclear, inhibiting therapeutic development. Available treatment options have limited efficacy and harbor safety concerns. Thus, there is a great need to clarify keloid pathomechanisms that may lead to novel treatments. In this study, we aimed to elucidate the profile of lesional and non-lesional keloid skin compared to normal skin. We performed gene (RNAseq, qRT-PCR) and protein (immunohistochemistry) expression analyses on biopsy specimens obtained from lesional and non-lesional skin of African American (AA) keloid patients compared to healthy skin from AA controls. Fold-change≥2 and false-discovery rate (FDR)<0.05 was used to define significance. We found that lesional versus normal skin showed significant up-regulation of markers of T-cell activation/migration (ICOS, CCR7), Th2- (IL-4R, CCL11, TNFSF4/OX40L), Th1- (CXCL9/CXCL10/CXCL11), Th17/Th22- (CCL20, S100As) pathways, and JAK/STAT-signaling (JAK3) (false-discovery rate [FDR]<0.05). Non-lesional skin also exhibited similar trends. We observed increased cellular infiltrates in keloid tissues, including T-cells, dendritic cells, mast cells, as well as greater IL-4rα^+^, CCR9^+^, and periostin^+^ immunostaining. In sum, comprehensive molecular profiling demonstrated that both lesional and non-lesional skin show significant immune alternations, and particularly Th2 and JAK3 expression. This advocates for the investigation of novel treatments targeting the Th2 axis and/or JAK/STAT-signaling in keloid patients.

## Introduction

Keloids are common, benign fibroproliferative overgrowths that manifest as exophytic, hyperpigmented lesions, extending beyond the original wound and result from dysregulated wound-healing secondary to skin injury. Keloids may recur, are associated with pain and pruritus, and are disfiguring (1). Numerous pathophysiologic processes have been investigated (1–3), including hypoxic (4, 5), mechanical tension (6, 7), and inflammatory etiologies (8–12). In addition, genetic studies have identified certain susceptibility loci, including inflammation-related genes, to be significantly associated with keloids (13–18). Yet, the pathogenesis still remains unclear, inhibiting therapeutic development. Moreover, current treatment options have limited efficacy and safety profiles and are associated with high recurrence rates of up to 100% (2, 19). Thus, there is a large unmet need to understand the pathomechanisms of keloids and identify novel therapeutics.

Recently, our group suggested a T-helper (Th)2-skewed pathogenesis of keloids by way of targeting Interleukin(IL)-4Rα with dupilumab, an FDA-approved biologic for moderate-to-severe atopic dermatitis (AD) (20). In this report, treatment of severe AD in an African American patient resulted in both AD improvement and shrinkage of his concomitant keloids (20). Furthermore, a limited analysis demonstrated increased mRNA expression of three Th2-related products (20), suggesting a possible Th2 involvement in mechanisms underlying pathologic fibrosis (21, 22). Clinical associations between keloids and other Th2-skewed diseases (e.g. AD (23) and asthma (24)) have also been reported. However, to our knowledge, a transcriptomic profiling of keloid skin lesions, that attempts to elucidate their immune alterations is lacking.

While a few gene expression studies have been performed, the majority focused on specific genes or limited microarray studies (25–32). Furthermore, these studies did not entirely characterize the inflammatory profile of keloids. Earlier cDNA microarray analyses primarily associated keloids with alterations of the extracellular matrix, growth factors, apoptosis-related molecules, and/or chondrogenic or osteogenic tissue differentiation (25–28). A more recent microarray profiling study analyzed differences in lesional versus non-lesional skin and reported bone and/or cartilage abnormalities but did not evaluate inflammatory pathways (29). Jumper et al. (30) used a laser capture micro-dissection approach, describing dysregulation of a few immune markers in keloid tissues (e.g. IL-13Rα1, IL-1β). Onoufriadis et al. (31) undertook an integrative mRNA and miRNA expression approach, focusing on pathway enrichment analysis and highlighting expression of mitogen-activated protein-kinase signaling pathway in keloid-prone individuals.

Due to a greater prevalence of keloids in individuals of African descent (19), we present a global RNA-seq profiling of skin biopsies obtained from both lesional and non-lesional skin of African American patients compared to healthy controls, complemented with additional validation using quantitative real-time PCR and immunohistochemistry. Our data show immune dysregulation, particularly Th2 and JAK3-skewing, along with Th1 and Th17/Th22 expression, in keloid lesions, extending to uninvolved skin, suggesting the potential for systemic therapies targeting these pathways in keloid patients.

## Materials and Methods

### Patient Characteristics and Sample Collection

Three African American patients (three females, mean age 47.3 years), with history of severe chronic keloids and no concomitant atopy or other comorbidities (29), and five healthy African American controls (two females, three males, mean age 39.8 years) were recruited under institutional review board-approved protocols. Biopsy specimens (6 mm) were collected from keloid lesions on the upper trunk. Non-lesional skin biopsies were obtained from a similar anatomic region, but at least 10 centimeters away from the keloid lesion. In one of the patients, a biopsy of an emerging keloid lesion was also obtained. Normal skin biopsy samples were collected from the trunk of healthy controls.

### RNA-Sequencing

RNA was extracted as previously described (33, 34). Libraries were generated using TruSeq Stranded mRNA Library Prep kit (Illumina). Next generation sequencing was performed on Illumina NovaSeq6000 (Illumina Inc., 100 cycles, single-read sequencing). Sample quality was assessed using FastQC. RNA-sequencing data was profiled by Illumina NovaSeq6000 to allow for more global analyses of genomic abnormalities. Data were pre-processed using standard pipeline incorporating quality control metrics, such as FastQC and MultiQC, sequence alignment based on STAR RNA-sequencing aligner, and sequencing reads assignment to genomic features by featureCounts and *voom*-transformed. A total of 4 keloid lesions obtained from 3 different patients and 6 healthy controls were included in the RNA-sequencing analysis. 

### Quantitative Real-Time Polymerase Chain Reaction

RNA was extracted for real-time polymerase chain reaction (RT-PCR) using the miRNAeasy Mini Kit (Qiagen, Hilden, Germany). Reverse transcription to complementary DNA (cDNA) from RNA was carried out using the High Capacity cDNA reverse transcription (Thermo Fisher). Pre-amplification was performed on all samples. Primers are listed in **Table S1**. Ten nanogram total RNA was used for PreAMP pool. *Rplp0* was used as endogenous control. Expression values were normalized to *Rplp0*.

### Immunohistochemistry

Immunohistochemistry was performed on frozen lesional and non-lesional skin sections from keloid patients and healthy skin from controls, using monoclonal antibodies as previously described (**Table S2**) (35, 36). Cell counts were quantified from representative sections in both the epidermis and dermis using ImageJ V1.42 software (National Institutes of Health, Bethesda, Maryland).

### Statistical Analysis

Analyses were performed using R-language (R-project.org) and Bioconductor Project packages (www.bioconductor.org). Gene expression profiles were modeled by linear models using R’s *lme* function. *limma* framework was used for RNA-sequencing data, and p-values were adjusted for multiple hypotheses using the Benjamini-Hochberg procedure. Fold changes (FCHs)>2.0 and false discovery rates (FDR)<0.05 were considered differentially expressed. We also evaluated a curated immune gene-subset (33, 34, 37) using P-values, due to the small sample size. Mean expressions are displayed in a heatmap, where unsupervised clustering was performed using Euclidean distance and average agglomeration criteria. PCR and immunohistochemistry data were log2 transformed and analyses were performed using Student’s t-test.

## Results

We performed RNA-seq global profiling (GSE158395), followed by qRT-PCR and immunohistochemistry, on lesional and non-lesional keloid skin samples obtained from three African American patients (mean age 47.3 years) with history of chronic (present for >10 years), large keloids, and no atopy or other comorbidities (29), and healthy skin samples obtained from five African American controls (mean age 39.8 years). In one of the patients, a biopsy of an emerging keloid lesion was also obtained. Patients did not have any treatments on their biopsied keloids. No significant differences in age or gender were detected between patients and controls.

### RNA-Seq Molecular Profiling Shows Immune Dysregulation With Significant Th2, Th1, Th17/Th22, and JAK3-Skewing in Keloid Lesions

Using criteria of fold-change (FCH)>2 and false discovery rate (FDR)<0.05 to define differentially expressed genes (DEGs), we detected 3,044 DEGs (1,437 up-regulated and 1,607 down-regulated) in lesional keloid versus normal skin, 2,929 DEGs between lesional and non-lesional skin, and few DEGs between non-lesional and normal skin, as depicted in a heatmap in **Figure S1** (**Table S3**).

**Figure 1:**
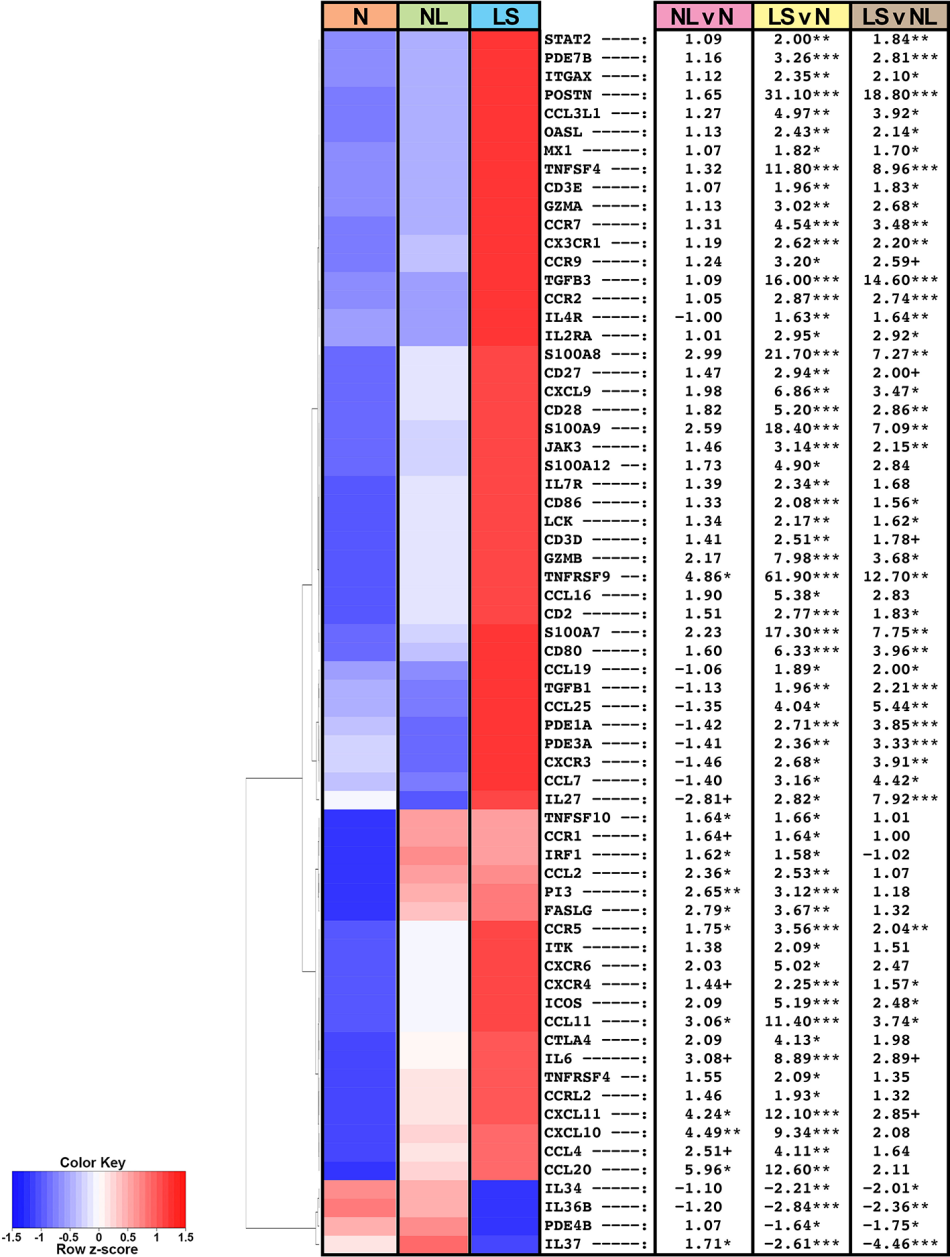
Summary heatmap of immune gene expression of multiple pathways in biopsied lesional and non-lesional keloid skin and normal skin from controls using RNA-seq. Table shows fold-changes in non-lesional versus normal (NL vs N), lesional versus normal (LS vs N), and lesional vs non-lesional (LS vs NL) skin. ***P < 0.001, **P < 0.01, *P < 0.05, ^+^P < 0.1. Red color denotes higher mean expression levels and blue color denotes lower mean expression levels. *LS*, lesional; *NL*, non-lesional; *N*, normal.

The top 50 DEGs in lesional versus normal skin comparison included products related to fibrosis and cartilage/bone-differentiation (ADAM12, ASPN, COL10A1, COL11A1, FBN2), previously reported to be dysregulated in keloids (**Table S3**) (20, 29). Periostin, implicated both in fibrosis as well as in Th2 inflammation (38–40), was among the top DEGs in lesional versus normal skin (FDR<0.05 for all; **Figure 1**, **Table S3**).

Among the significantly modulated genes in keloid lesions compared to normal skin also included markers related to various immune components, as depicted in a heatmap of a curated immune gene-subset (**Figure 1**) (33, 34, 37). In keloid lesions, we observed significant increases in measures of T-cells (CD2, CD3, CD28), T-cells/NK-cells/T-cell activation and migration (ICOS, LCK, GZMA/B, CCR7, TNFRSF9), and cellular infiltrates, such as dendritic cell markers CD80 and CD86 (FDR<0.05; **Figure 1**, **Table S3**). Measures of innate immunity (IL-6), and multiple immune pathways, including the Th2- (IL-4R, CCR5, CCL11, TNFSF4/OX40L), Th1- (CXCL9/CXCL10/CXCL11, OASL), and Th17/Th22- (CCL20, PI3, S100A7/8/9) pathways were also significantly up-regulated (FDR<0.05; **Figure 1**, **Table S3**).

Furthermore, keloid lesions displayed significant up-regulation of JAK/STAT signaling molecule, JAK3, as compared to normal skin (FDR<0.05; **Figure 1**, **Table S3**). Markers related not only to fibrosis but also Tregs (TGFβ1 and TGFβ3) (41, 42), were highly increased as well (FDR<0.05). Both CCR9, the homing receptor for the small intestine and lung (43, 44), and its ligand CCL25, displayed increased expression in keloid tissues (FDR ≤ 0.1). The CCR9/CCL25 axis has been shown to induce cellular recruitment in early allergic asthma (44). Negative immune regulators, IL-34 (45) and IL-37 (34), were significantly reduced in lesional keloid versus normal skin (FDR<0.05; **Figure 1**, **Table S3**).

Similar dysregulation of these immune axes was observed between non-lesional and normal skin comparisons, albeit these did not attain significance by FDR, however some achieved significance using criteria of P<0.05 (e.g. CCL11, CCR5, CXCL10/11, CCL20) (**Figure 1**, **Table S3**).

### qRT-PCR Validates and Expands RNA-Seq Data

**Figure 2:**
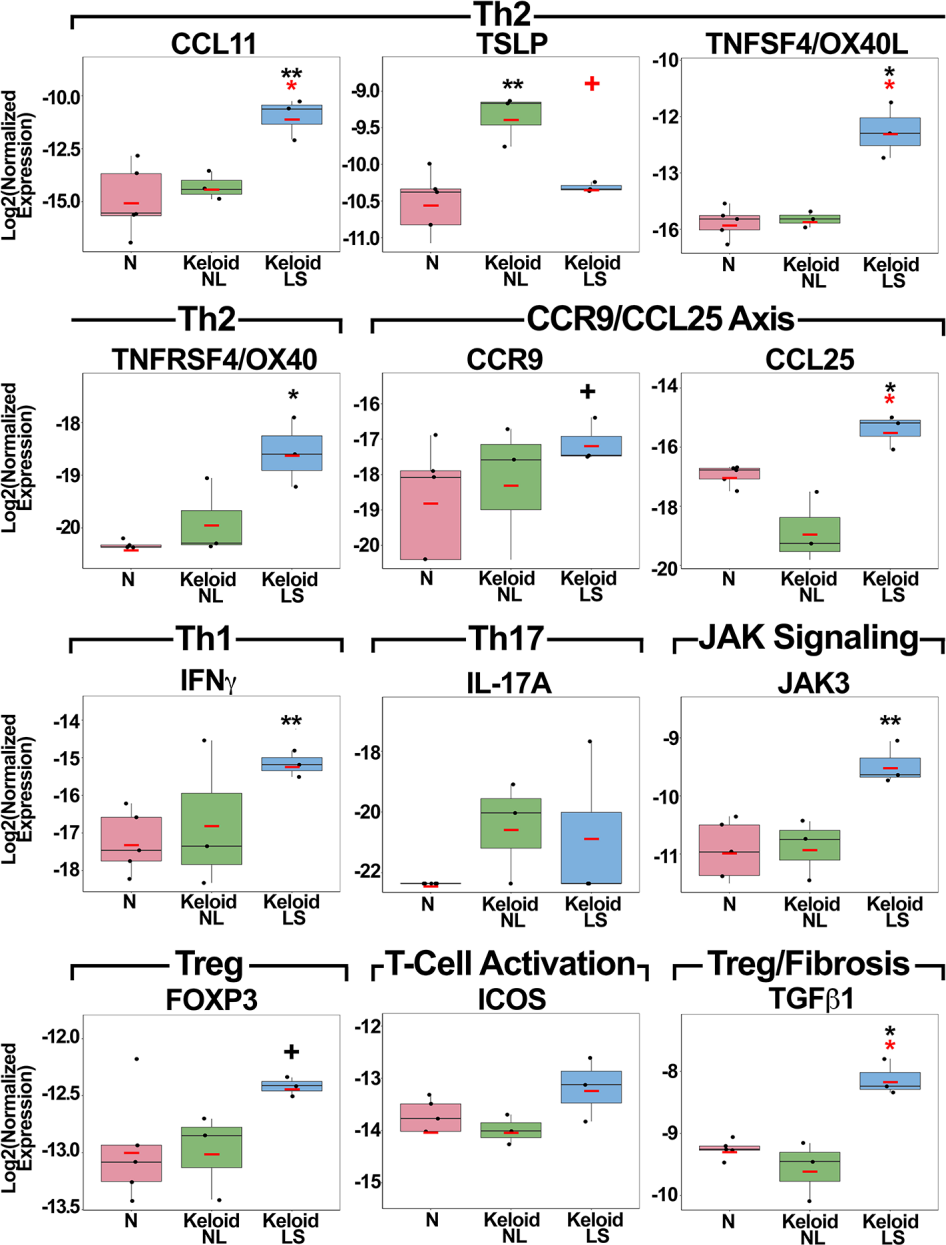
Fold-changes of immune mediators in lesional and non-lesional skin of keloid patients, as well as normal skin, as measured by quantitative real-time PCR. Red bar represents mean. *Black symbols*: significance of comparison to normal skin*; red symbols:* significance of comparison between lesional and non-lesional skin. **P < 0.01, *P <0.05, ^+^ *P* < 0.1. *PCR*, polymerase chain reaction; *LS*, lesional; *NL*, non-lesional; *N*, normal.

qRT-PCR was performed to validate as well as evaluate key immune molecules that are often below detection limits on RNA-seq (34, 46) (**Figure 2**). Th2 (CCL11, TSLP, TNFSF4/OX40L, TNFRSF4/OX40) markers showed significant up-regulation in lesional or non-lesional versus normal skin (P<0.05; **Figure 2**). Expression of CCR9 and its ligand CCL25 showed significant, or trending toward significant, up-regulation in lesional versus normal skin (P<0.05 for CCL25; **Figure 2**). JAK3 was also highly expressed in keloid lesions compared to normal skin (P<0.01; **Figure 2**).

While the main Th1 cytokine, IFN*γ*, showed significant increases in keloid lesions (P<0.01), IL-17A, the Th17 cytokine, only showed a trend for increased expression in both lesional and non-lesional versus normal skin (**Figure 2**). Treg markers, FOXP3 and TGFβ1, and T-cell activation measure ICOS, were up-regulated in lesional keloid versus normal skin (P<0.05 for TGFβ1; **Figure 2**). We also evaluated an emerging keloid lesion and obtained similar gene expression results (**Figure S2**).

### Immunohistochemistry Shows Marked Cellular Infiltrates in Keloids

**Figure 3:**
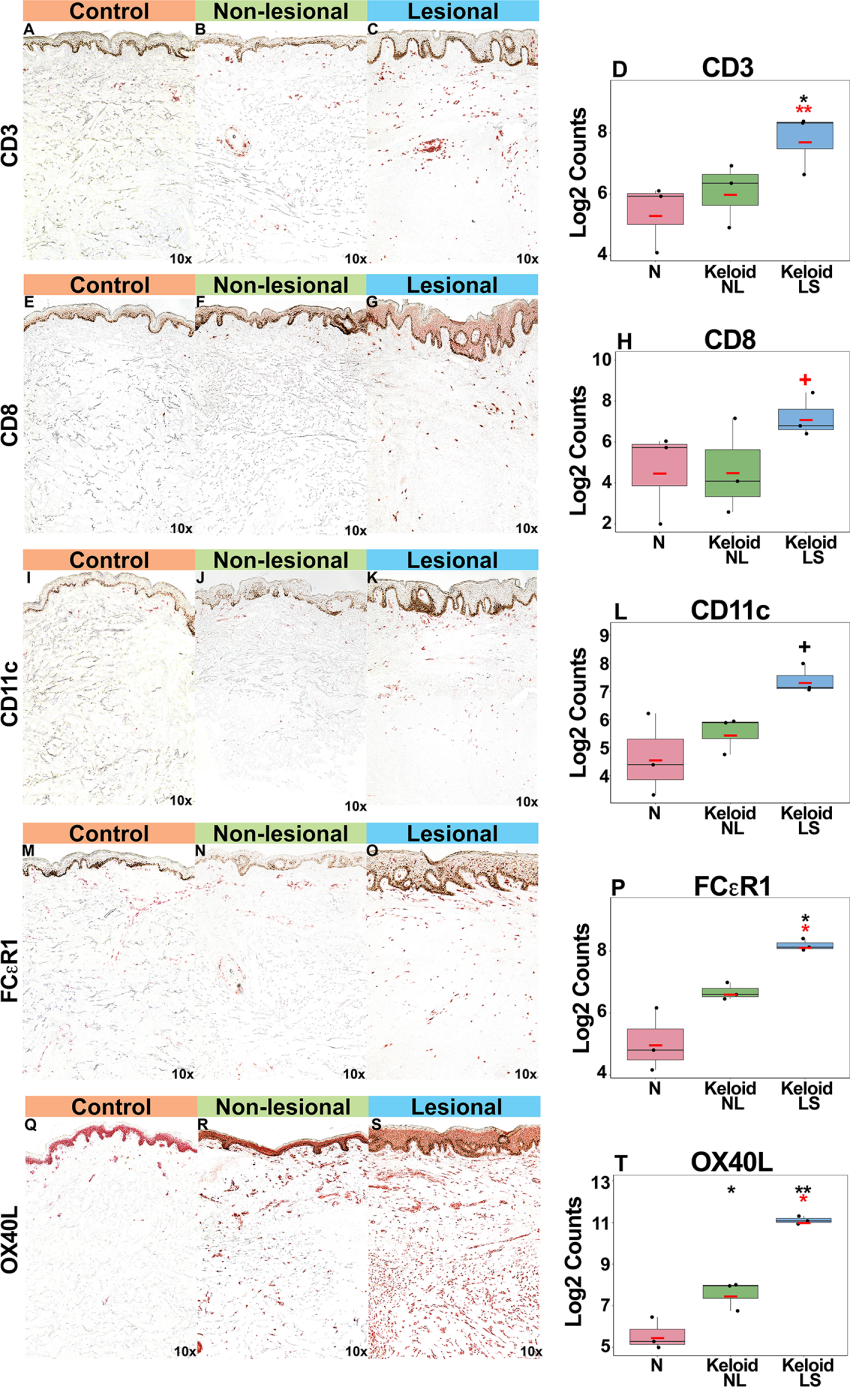
Representative immunohistochemistry images and corresponding cell count quantification of CD3^+^ T-cells **(A–D)**, CD8^+^ T-Cells **(E–H)**, CD11c^+^ dendritic cells **(I–L)**, FCεR1^+^ dendritic cells **(M–P)** and OX40L^+^ dendritic cells **(Q–T)** in normal skin from controls, and lesional and non-lesional skin of keloid patients, viewed at 10x magnification. Red bar represents mean. *Black symbols*: significance of comparison to normal*; red symbols:* significance of comparison between lesional and non-lesional skin. **P < 0.01, *P < 0.05, ^+^ *P* < 0.1. *LS*, lesional; *NL*, non-lesional; *N*, normal.

Gradual increases were observed for multiple cellular infiltrate measures, such as T-cells (CD3^+^, CD8^+^) and dendritic cells (CD11c^+^), including dendritic cell infiltrates that are characteristic to AD, such as markers typifying “atopic” dendritic cells (OX40L^+^, FCεR1^+^) (33, 47, 48) (P<0.05 for CD3^+^, FCεR1^+^, OX40L^+^ in lesional versus normal skin comparisons; **Figures 3A–T**). OX40L^+^ immunostaining was also significantly increased in non-lesional keloid versus normal skin (P<0.05; **Figures 3Q–T**).

**Figure 4:**
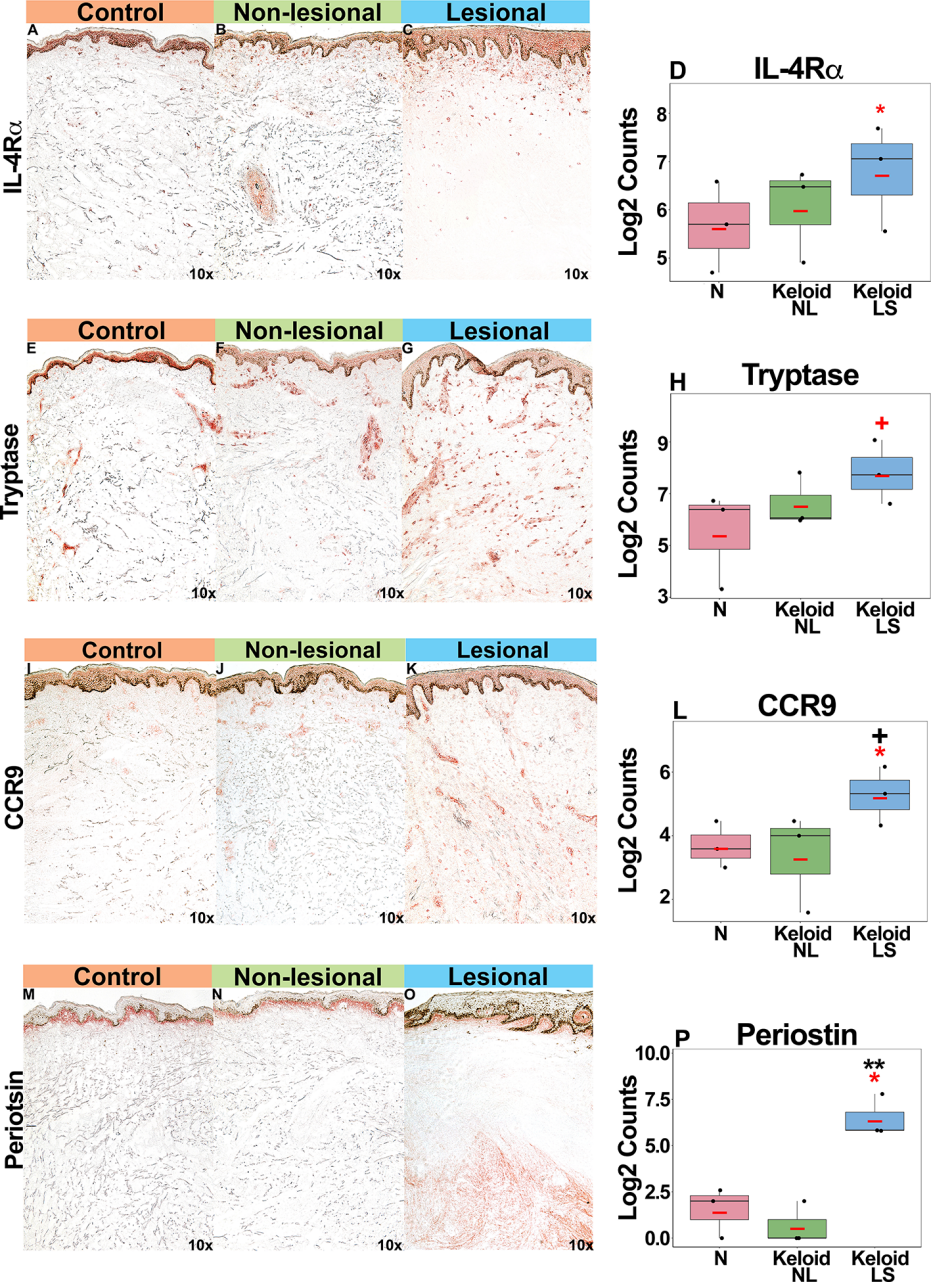
Representative immunohistochemistry images and corresponding cell count quantification of IL-4Rα^+^ cells **(A–D)**, tryptase^+^ mast cells **(E–H)**, CCR9^+^ cells **(I–L)** and periostin^+^ cells **(M–P)** in normal skin from controls, and lesional and non-lesional skin of keloid patients, viewed at 10x magnification. Red bar represents mean. *Black symbols*: significance of comparison to normal*; red symbols:* significance of comparison between lesional and non-lesional skin. **P < 0.01, *P < 0.05, ^+^ *P* < 0.1. *LS*, lesional; *NL*, non-lesional; *N*, normal.

Supporting our mRNA expression data, we noted increased IL-4Rα^+^ immunostaining in both lesional and non-lesional keloid skin as compared to normal skin (P<0.05 for lesional versus non-lesional skin comparison; **Figures 4A–D**). Mast cells (tryptase^+^), associated with atopy (49, 50), were increased in keloid lesions (P<0.1 for lesional versus non-lesional comparison; **Figures 4E–H**). Trending toward significantly greater CCR9^+^ immunostaining was noted in lesional compared to normal skin (P<0.1 **Figures 4I–L**). Finally, we observed significantly greater periostin immunostaining, concentrating in the lower dermis of keloid lesions, as compared to non-lesional and normal skin (P<0.05; **Figures 4M–P**). We also evaluated an emerging keloid lesion and obtained similar cellular infiltrates (**Figure S3**).

## Discussion

Our study combines gene and protein expression analyses, broadly characterizing the molecular and cellular phenotype of lesional and non-lesional keloid skin, highlighting the possible contribution of immune abnormalities to its pathogenesis. While various etiologies for keloid formation have been investigated, the pathogenesis remains to be clearly elucidated, hindering the development of novel therapeutics. Global RNA-seq profiling, as successfully performed in other inflammatory skin diseases, such as AD and psoriasis (34, 51), may uncover further insights into disease mechanisms, and possibly lead to the development of newer, immune-based treatments.

In light of the role of inflammation in normal wound healing, a dysregulated immune component can contribute to an abnormal wound repair process (8, 9, 52). However, to our knowledge, the role of inflammation in the pathogenesis of keloids has not been adequately investigated. Published studies investigating an immune component have largely focused on specific keloidal cell cultures or specific genes. One report identified up-regulation of the proinflammatory cytokines, IL-1α, IL-1β, IL-6, and TNFα, in keloid fibroblasts (10). Our group recently examined a few Th2 markers in keloids (20), while another study found increases for the IL-17/IL-22-induced products, S100A7 and hBD2 (53). Additionally, dysregulation of the IL-17/IL-6 axis (11) and/or JAK/STAT-signaling (54, 55) have also been investigated. The present study expands on prior investigations and provides a comprehensive molecular profiling of keloids, identifying a significant immune component in both lesional and non-lesional skin, as well as linking keloids with fibrosis and cartilage/bone-differentiation.

Our results associate keloid tissues with an inflammatory milieu representing multiple T-helper pathways, including the Th2 (e.g. IL-4R, CCL11, TSLP, TNFSF4/OX40L, TNFRSF4/OX40), Th1 (e.g. IFN*γ*, CXCL10/11), Th17/Th22 (e.g. PI3, CCL20, S100As) axes, as well as the JAK/STAT signaling molecule JAK3. The up-regulation of Th17 markers expands upon prior reports demonstrating a role of Th17 mediators in keloids (11, 55). Additionally, our results suggest that, similar to other fibrotic diseases (56), the Th1 axis is also involved to keloid pathogenesis. Of note, IL-6 which is associated with innate immunity (57), is highly up-regulated in our study and has also been shown to drive a chronic pro-fibrotic state *via* a Th1-mediated response (58).

In the present study, we observed a significant Th2 signature in keloids as well as increased cellular infiltrates associated with the Th2 microenvironment, such as tryptase^+^ mast cells (12, 49, 50), markers of dendritic cells characteristic to atopic dermatitis (AD) (OX40L^+^ and FCεR1^+^) (33, 47, 48), IL-4Rα^+^ cells, and periostin^+^ cells. In human fibroblasts, it has been found that IL-4 and IL-13 increased TGF-β signaling and enhanced fibrosis *via* periostin (59). Periostin is a matricellular protein that plays important physiologic and pathogenic roles in skin fibrosis (60, 61) and serves as a biomarker for several known Th2-associated diseases (e.g. AD, asthma, nasal polyps, systemic sclerosis) (38, 39, 62, 63). Its significantly increased upregulation in keloids may represent an important link between immune responses and fibrosis. In fact, one study found periostin to be expressed by a novel subpopulation of Th2-associated fibroblasts in AD (64). These data implicate a cross-talk between Th2 immune mechanisms and fibrosis that is also likely relevant in keloid pathogenesis.

Further reinforcing the concept of immune dysregulation in driving fibrotic processes in keloids is the increased T-cell expression, including Th2-related markers, in several fibrotic conditions, such as frontal fibrosing alopecia and scleroderma (65, 66). *In vitro* studies have also demonstrated the profibrotic effects of the type 2 cytokines IL-4 and IL-13 (22), including increased collagen production in keloid fibroblasts after IL-13 stimulation (67). These studies strengthen our hypothesis that immune dysregulation, possibly Th2 driven, plays a significant role in keloid formation. Additionally, we found increased expression of CCR9 and its receptor CCL25, which are involved in inflammatory cell recruitment in early allergic asthma (44). Taken together, our results highlight commonalities between keloids and other atopic disorders such as AD and asthma (68), as well as those that exhibit exophytic growth (e.g. nasal polyps) (69, 70). Keloids also display commonalities with connective tissue diseases associated with excessive extracellular matrix deposition, such as scleroderma (65).

The up-regulation of T-cell related genes in non-lesional skin compared to normal skin (e.g. CCL11, CCR5) highlight that the seemingly normal skin of keloid patients is in fact abnormal and predisposed to formation of keloids. These data are also supported by the clinical observation that individuals who develop keloids are prone to future keloid formation after skin wounding (19). These incipient findings in non-lesional skin are important as it might mean that patients with significant keloids may necessitate systemic treatment to target not only lesional but also non-lesional skin to prevent future recurrence of keloids.

While studies in blood can clarify the presence of systemic activation in keloid patients, our results, which demonstrate immune dysregulation extending beyond skin lesions to uninvolved skin, advocate for the investigation of systemic treatments for keloids, such as those targeting the Th2 axis. The potential clinical utility of anti-IL-4Rα antagonism (20) is encouraged by the significant overlap observed between dupilumab-regulated markers in AD and those that are dysregulated in keloids, including markers of the Th2 axis, as well as cellular infiltrates (CD3^+^, CD11c^+^, FCεR1^+^), T-cell activation/migration (GZMB, ICOS, CCR7), and the Th17/Th22 (PI3, S100As) pathway (37, 71). Antagonism of the OX40-TSLP axis, which is known to perpetuate Th2 activation in both AD and asthma (72–74), could also be explored for keloids. GBR830 and KHK4083 are anti-OX40 monoclonal antibodies in clinical trials (75, 76). In one analysis (75), GBR830 significantly decreased measures that are also upregulated in keloid tissues in the present study, including OX40^+^ T-cells, OX40L^+^ dendritic cells, and Th2- (CCL11), Th1- (IFN*γ*, CXCL10), and Th17/Th22- (S100A9/12) related markers. Given the significantly increased expression of TSLP in non-lesional keloid skin, exploration of tezepelumab, an anti-TSLP monoclonal antibody (77), may also be warranted.

Furthermore, biologics targeting IL-6 signaling, such as tocilizumab, sarilumab, and siltuximab, could be explored (78). Indeed, our data showing significant IL-6 up-regulation in keloids expands on previous studies that demonstrated elevated IL-6 expression in keloidal fibroblasts (79, 80). Ghazizadeh et al. (80) further showed that inhibition of IL-6 or IL-6Rα in keloid fibroblasts resulted in a reduction of collagen synthesis, underscoring the potential utility of IL-6 antagonism for this disease.

In contrast to the aforementioned biologics, Janus kinase (JAK) inhibitors comprise a class of broad-acting small molecules. The JAK/STAT signaling and spleen tyrosine kinase (SYK) pathways are implicated in numerous autoimmune and inflammatory diseases (e.g. AD, psoriasis, alopecia areata) (81), modulating a range of immune responses, including the Th2 (IL-4, IL-13, CCL18), Th1 (IFN*γ*), and Th17/Th22 (CCL20, S100As) pathways (82). Preliminary studies evaluating inhibition of JAK/STAT signaling in human keloid fibroblasts (54, 55), and in a humanized keloid animal model (83), have demonstrated promising results. There are several JAK inhibitors in clinical development showing efficacy for various dermatologic disorders (81, 82, 84) and could be a therapeutic strategy worth investigating in keloids.

This is a preliminary study with a few limitations, including a small sample size. While future studies need to evaluate larger cohorts, we were still able to obtain large differences in lesional and non-lesional skin versus normal skin, as well as a significant signal after adjusting for multiple hypothesis testing by false-discovery rate using a stringent cut-off for significance. Our study group only included African American patients, representing a greater prevalence of keloids in this ethnicity (1, 19). Additional studies should evaluate keloids in other races and ethnicities, such as in Hispanics, Asians, and Caucasians (1, 19). Finally, as our study raises the hypothesis of possible immune-based treatments for keloids, this needs to be proven in future proof of concept studies and clinical trials.

In sum, the current genomic and cellular profiling study of keloids provides a comprehensive molecular fingerprint of the immune alterations in lesional and non-lesional keloid skin compared to normal skin. Our results reveal broad immune dysregulation, including up-regulation of markers of the Th1 and Th17/Th22 axes as well as significant Th2 and JAK3 expression in keloid lesions, extending to uninvolved skin, suggesting the potential use of systemic therapeutics. Future clinical trials for keloid patients may include those targeting the Th2 axis, inhibitors of the TSLP-OX40 axis, antagonists of JAK/STAT-signaling, or IL-6 inhibition.

## Data Availability Statement

The original contributions presented in the study are publicly available. This data can be found here: https://www.ncbi.nlm.nih.gov/geo with accession number GSE158395.

## Ethics Statement

The studies involving human participants were reviewed and approved by The Rockefeller University IRB. The patients/participants provided their written informed consent to participate in this study.

## Author Contributions

JW contributed to the study design, data analysis, data interpretation, and writing the manuscript. ED, ME, and AG contributed to the histology stainings, data interpretation, and writing the manuscript. IC, NZ, and YE contributed to the experiments. AP contributed to the study design, data analysis, data interpretation and writing the manuscript. JK edited and improved the manuscript. EG-Y contributed to the study and experimental design, data interpretation, and writing the manuscript. All authors contributed to the article and approved the submitted version.

## Conflict of Interest

EG-Y is an employee of Mount Sinai and has received research funds (grants paid to the institution) from: Abbvie, Celgene, Eli Lilly, Janssen, Medimmune/Astra Zeneca, Novartis, Pfizer, Regeneron, Vitae, Glenmark, Galderma, Asana, Innovaderm, Dermira, UCB. EG-Y is also a consultant for Sanofi Aventis, Regeneron, Stiefel/GlaxoSmithKline, MedImmune, Celgene, Anacor, AnaptysBio, Dermira, Galderma, Glenmark, Novartis, Pfizer, Vitae, Leo Pharma, Abbvie, Eli Lilly, Kyowa, Mitsubishi Tanabe, Asana Biosciences, and Promius. JK has received research support (grants paid to his institution) and/or personal fees from Pfizer, Amgen, Janssen, Lilly, Merck, Novartis, Kadmon, Dermira, Boehringer, Innovaderm, Kyowa, BMS, Serono, BiogenIdec, Delenex, AbbVie, Sanofi, Baxter, Paraxel, Xenoport, and Kineta. AP, NZ, and ED are employees of Mount Sinai.

The remaining authors declare that the research was conducted in the absence of any commercial or financial relationships that could be construed as a potential conflict of interest.

## Abbreviations

AD, atopic dermatitis; DC, dendritic cell; FCH, fold-changes; FDR, false discovery rate; IL, interleukin; qRT-PCR, quantitative real time polymerase chain reaction; RNA-seq, RNA-sequencing; Th, T-helper; Treg, T-regulatory cell.
