# Supplementary material for: RNA Sequencing Keloid Transcriptome Associates Keloids With Th2, Th1, Th17/Th22, and JAK3-Skewing
Source: Front Immunol. 2020 Nov 23;11:597741. doi: 10.3389/fimmu.2020.597741 (PMC7719808; doi:10.3389/fimmu.2020.597741)
Supplement: Supplementary file 2 [file Table_1.docx]

| TABLE S1. Primers and Probes for qRT-PCR | |
| --- | --- |
| Marker | **Probe** |
| CCR9 | Hs01890924_s1 |
| CCL25 | Hs00608373_m1 |
| ICOS | Hs00359999_m1 |
| IFNG | Hs00989291_m1 |
| CCL11 | HS00237013_m1 |
| TSLP | Hs00263639_m1 |
| TNFSF4 | Hs00182411_m1 |
| TNFRSF4 | Hs00937194_g1 |
| IL-17A | Hs00174383_m1 |
| JAK3 | Hs00169663_m1 |
| FOXP3 | Hs01085834_m1 |
| TGFB1 | Hs00998133_m1 |
