# Supplementary material for: RNA Sequencing Keloid Transcriptome Associates Keloids With Th2, Th1, Th17/Th22, and JAK3-Skewing
Source: Front Immunol. 2020 Nov 23;11:597741. doi: 10.3389/fimmu.2020.597741 (PMC7719808; doi:10.3389/fimmu.2020.597741)
Supplement: Supplementary file 3 [file Table_2.docx]

| TABLE S2. Antibodies for Immunohistochemical Analyses | | | | | |
| --- | --- | --- | --- | --- | --- |
| Name | **Manufacturer** | **Species** | **Clone** | **Isotype** | **Dilution** |
| CD3 (SK7) | BD Biosciences | Mouse | SK7 | IgG1 | 1:100 |
| CD8 | BD Biosciences | Mouse | HIT8a | IgG1 | 1:100 |
| CD11c | BD Biosciences | Mouse | B-ly6 | IgG1 | 1:100 |
| IL4Rα | R&D Systems | Mouse | 25463 | IgG2A | 1:300 |
| FCεR1 (AER-37) | NovusBiologicals | Mouse | CRA1 | IgG2b | 1:100 |
| Tryptase | GeneTex | Mouse | AA1 | IgG1 | 1:200 |
| Periostin | Novus Biologicals | Mouse | OTI2B2 | IgG2a | 1:500 |
| OX40L | R&D Systems | Mouse | 159403 | IgG1 | 1:50 |
| CCR9 | R&D Systems | Mouse | 112509 | IgG2a | 1:100 |
|  | | | | | |
