# Supplementary material for: RNA Sequencing Keloid Transcriptome Associates Keloids With Th2, Th1, Th17/Th22, and JAK3-Skewing
Source: Front Immunol. 2020 Nov 23;11:597741. doi: 10.3389/fimmu.2020.597741 (PMC7719808; doi:10.3389/fimmu.2020.597741)
Supplement: Supplementary file 4 [file Table_3.docx]

**Supplementary Figure 1**

Heatmap of all differentially expressed genes by RNA-seq in normal, non-lesional, and lesional keloid skin. *LS,* lesional; *NL*, non-lesional; *N*, normal

**Supplementary Figure 2**

Fold-changes of immune mediators in lesional and non-lesional skin of keloid patients, including the emerging keloid, as well as normal skin, as measured by quantitative real-time PCR. Red bar represents mean. *Black symbols*: significance of comparison to normal skin*; red symbols:* significance of comparison between lesional and non-lesional skin. ***P*<0.01, **P*<0.05, +*P*<0.1. *PCR,* polymerase chain reaction; *LS,* lesional; *NL*, non-lesional; *N*, normal

**Supplementary Figure 3**

Cell count quantification of immunohistochemistry staining in lesional and non-lesional skin of keloid patients, including the emerging keloid, as well as normal skin. Red bar represents mean. *Black symbols*: significance of comparison to normal*; red symbols:* significance of comparison between lesional and non-lesional skin. ***P*<0.01, **P*<0.05, +*P*<0.1. *LS,* lesional; *NL*, non-lesional; *N*, normal
