# Supplementary figures and images for: RNA Sequencing Keloid Transcriptome Associates Keloids With Th2, Th1, Th17/Th22, and JAK3-Skewing
Source: Front Immunol. 2020 Nov 23;11:597741. doi: 10.3389/fimmu.2020.597741 (PMC7719808; doi:10.3389/fimmu.2020.597741)

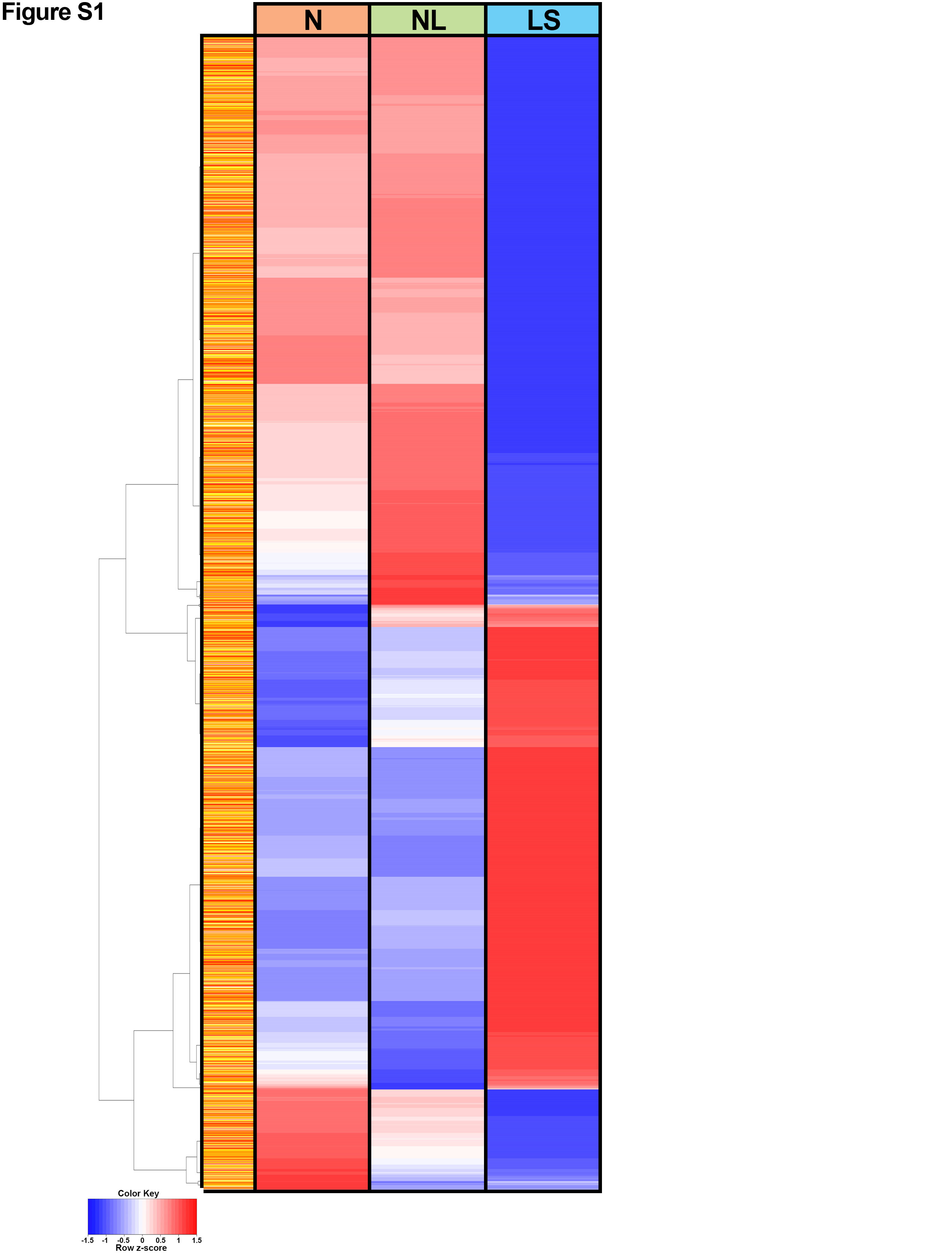

Supplement: Supplementary file 5 [file Image_1.jpeg]
